# Psychosocial risk factors for injury in performing artists: A scoping review of screening and assessment instruments

**DOI:** 10.1371/journal.pone.0322971

**Published:** 2026-02-24

**Authors:** Róisín Cahalan, Caoimhe Barry Walsh, Orfhlaith Ni Bhriain, Breandán de Gallaí, Hannah Fahey de Brún, Michele Pye, Rose Schmieg

**Affiliations:** 1 School of Allied Health, University of Limerick, Limerick, Ireland; 2 Physical Activity for Health Research Centre, University of Limerick, Limerick, Ireland; 3 Irish World Academy of Music and Dance, University of Limerick, Limerick, Ireland; 4 Shenandoah University, Division of Athletic Training, Shenandoah University, Virginia, United States of America; 5 Department of Physical Therapy, College of Nursing and Allied Health Science, Howard University, Washington, District of Columbia, United States of America; Università degli Studi di Milano: Universita degli Studi di Milano, ITALY

## Abstract

The performing arts is a diverse collection of disciplines, sharing an elevated risk of injury related to an array of biopsychosocial risk factors. While screening for physical risk factors is common practice, and largely routine, psychosocial screening for injury in performance artists (PAs) is less well established. This scoping review aimed to systematically map the array of instruments used to screen for, or assess, psychosocial risk factors for injury in non-recreational adult performing artists (PA)s. Additionally, psychometric evaluations of each instrument in PA cohorts were reported where available. This scoping review was conducted in accordance with the Joanna Briggs Institute Evidence Synthesis guidelines. Twelve databases relating to performance, health, medicine, kinesiology, and sport were searched. Studies that investigated associations between psychosocial factors and injury in non-recreational (professional, pre-professional, full-time collegiate students, elite competitive) adult PAs were eligible. Fifty-one studies of 7,457 participants met the inclusion criteria (musicians: n = 4,505 (60.5%); dancers: n = 2,680 (35.9%); vocalists n = 225 (3.0%); circus performers: n = 47 (0.6%)). Most participants were professional PAs (n = 4,547 (61.0%)), followed by collegiate PAs (n = 1,424 (19.1%)), and mixed professional, pre-professional, elite competitive, and collegiate cohorts (n = 1,486 (19.9%)). Forty-five different psychosocial factors were identified across 90 distinct instruments. Stress, anxiety, depression and perfectionism were the factors most frequently investigated. Stress was commonly reported across all PA cohorts. The reliability of just 19 (21%) instruments was established for the target cohort. Many other instruments were valid/reliable in patient, sporting or general population cohorts, but untested in PAs. A common link between many psychosocial risk factors and injury in non-recreational adult PAs was identified. Screening programmes should incorporate comprehensive evaluations of these factors. Instruments appropriate for the cohort investigated should be used where available. The development and/or validation of instruments for use across all PAs for common risk factors should be considered.

## Introduction

The performing arts encompass diverse disciplines where artists use their voices, bodies, or inanimate objects to convey artistic expression [1]. This includes, but is not limited to, music, dance, song, circus and theatre arts. Although predominantly aesthetic activities, many performing artists (PAs) exist at the nexus between the artist and the athlete. Significant physical and psychological ability and reserves are required to withstand the rigours of the highly repetitive nature of performance training and the threat of pain and injury [2].

The occurrence of injury across various disciplines in the performing arts is well documented. A recent review of dancers from various genres and at different levels (recreational, student elite and professional dancers) reported that injury in dance remains a concerning issue. This is further exacerbated by a paucity of pertinent of high-level evidence and inconsistency in research methods [3]. Injury incidence in dancers has been found to range from 0.16 to 4.6 per 1000 hours of exposure [4,5], with point and period prevalence of 54.8% [6] and 280% [7] respectively, reported in the literature. Similarly in musicians, high levels of pain, weakness and other musculoskeletal disorders have been recorded [8,9]. A recent systematic review of adult musicians reported a lifetime injury prevalence of 46–90% [10]. In vocalists, phonotrauma is largely understood to stem from cumulative vocal fold tissue damage and/or response to persistent tissue inflammation [11]. An overall career prevalence of self-reported dysphonia (hoarse, raspy, strained voice) in singers of 46.09% has been reported in the literature [12]. Furthermore, it is estimated that up to 29% of attendees at voice clinics in the United States are professional vocalists, even though they represent less than 1% of the national work force [13].

Interestingly, there is considerable overlap in many of the identified causes of injury across the performing arts. Much of the research in this area focuses on physical causes of injury, with factors including overtraining, excessive load and under-recovery identified in studies across music, song and dance respectively [14–16]. A study exploring injury in dancers and musicians identified numerous factors including biomechanical issues, poor ergonomics and suboptimal technique as key issues driving injury in both groups [17]. Prior injury is also noted as an important risk factor for future injury across many areas of the performing arts [18–20].

There is also a growing understanding of the association between psychosocial factors and injury in the performing arts [21–23]. A systematic review of professional and pre-professional instrumentalists identified an association between an increased level of musculoskeletal disorders, and elevated levels of stress and performance anxiety [19]. Similarly, emotional exhaustion, poor self-efficacy and fatigue were found to be related to an increased injury risk in a cohort of circus artists [24]. Likewise, in vocalists, an association between personality traits/facets related to happiness, dominance and caution, and phonotrauma were reported [15]. A multitude of factors including stress, psychological distress, disordered eating, poor coping, suboptimal sleep, personality, and social support have also been found to be associated with increased injury risk or adverse injury outcomes in dancers [25]. Additionally, the experience of being seriously injured can be devastating for the PA, whose identity is intimately entwined with their craft [26,27]. The extent to which the PA experiences these challenges to their identity, and repercussions therein are known to be impacted by many factors including personality traits, coping strategies, and quality of social support [28].

The use of appropriate screening or assessment instruments is essential to identify the psychosocial risk factors which may arise in PAs. Such instruments allow for the systematic assessment of psychosocial constructs that are otherwise difficult to quantify, enabling early identification of at-risk individuals and informing targeted interventions. Recent efforts to develop a holistic PA screening instrument, including physical and psychological evaluations specific to individual cohorts, are promising [29], but large-scale studies are lacking. There are a range of tools that have been developed specifically for psychosocial risk evaluation in cohorts within the performing arts, but it is unclear if these instruments may be adapted for the broader PA community. For example, the Kenny Music Performance Anxiety Inventory (KMPAI) has shown promise in capturing relevant psychosocial dimensions in musicians [30] and opera singers [31], though further exploration and validation may be required across performing arts disciplines as a whole. Similarly, the widely used Hospital Anxiety and Depression Scale (HADS), which has been found to be appropriate for evaluating markers of anxiety and depression in dancers [32], is untested in other PAs. Furthermore, in the absence of bespoke tools, researchers and clinicians may utilise instruments that have been developed for use in other cohorts, such as patient groups or the general public. However, given the aforementioned unique traits and experiences of PAs, the accuracy and utility of untested instruments in this cohort are questionable.

It is our contention that, despite the heterogeneous physical demands of PAs, the psychosocial profile of injured PAs is similar across disciplines. There may, therefore, be an opportunity for the development of common instruments to screen for and identify psychosocial injury risk across disciplines. An important initial step is to catalogue both the instruments used for this purpose, the specific PA cohorts they have been used with, and the psychosocial risk factor that they evaluate. To our knowledge, there has been no previous study which has undertaken this exercise. The objective of this scoping review, therefore, was to systematically map any instruments used to screen or assess psychosocial risks for injury in non-recreational PAs. A secondary objective was to report whether the instrument had previously been evaluated psychometrically in the cohort of interest.

## Methods

### Protocol and registration

Owing to the heterogeneity of the available literature, a scoping review was identified as the most appropriate methodology to address the aims of this study [33]. This scoping review was conducted in accordance with the Joanna Briggs Institute (JBI) Evidence Synthesis guidelines [34] and Preferred Reporting Items for Systematic Reviews and Meta-Analyses extension for Scoping Reviews (PRISMA-ScR) [35]. A scoping review protocol in accordance with the Joanna Briggs Institute was registered on the Open Science Framework (https://osf.io/sjntx/).

### Inclusion criteria

- Original peer-reviewed research, and clinical practice guidelines reporting screening, assessment or evaluation instruments (including surveys, questionnaires) of psychosocial factors associated with injury in PAs, were included. For conciseness, the term “injury” will be used in this text, but references to pain, physical disorders, wounds and ailments in the literature were also included in search parameters. (Table 1). Pertinent systematic reviews in the area were screened for studies meeting the inclusion criteria.
- Studies involving adult (18 years and over) dancers, musicians, singers, circus artists, or other physical performance (e.g., theatre actors).
- Non-recreational PAs, including professional, pre-professional, full-time University (or equivalent) performing arts students, or otherwise described elite performers (such as competitive at highest possible level in that genre) were included. Studies of mixed (recreational and non-recreational) artists were only included if data pertaining to non-recreational performing artists could be extracted.
  - English language, human research studies published from the date of inception of the database, were considered. Databases were searched from inception to ensure an extensive and thorough search of the literature in this novel area.

**Table 1:** Key concepts informing search strategy.

Concept A Risk	Concept B Activity	Concept C Population	Concept D Injury	Concept E Measurement
Psycho-social	Performing artists	Adult	Musculoskeletal injury	Instrument or tool
Stress	Dance	Professional	Pain	Survey
Anxiety	Vocalist/singer	Pre-professional	Wound	Questionnaire
Catastrophis(z)ing	Musician/ instrumentalist	Full-time Student	Disorder	Assessment
Depression	Theatre actor	Elite	Ailment	Screening
Cognitive/mental	Circus			

### Exclusion criteria

- Studies including instruments which assessed/measured solely physical factors including load and physical fatigue.
- Instrument was not used to assess a relationship/association with injury.
- Qualitative (non-instrument based) reports of psychosocial issues/risks.
- Studies of recreational PAs.
- Study protocols as associations between variables are not recorded, case reports, abstracts, conference proceedings and other secondary research studies.
- Studies of PAs under the age of 18 years.

### Information sources

Database searches were conducted in November 2024 and limited to the following databases: Web of Science; EMBASE; Cochrane Database of Systematic Reviews; EBSCO (CINAHL Ultimate, MEDLINE, SPORTDiscus, PsycINFO); PubMed; Elsevier (ScienceDirect; Scopus); ProQuest Performing Arts Periodical Database, Dissertations); SAGE; JSTOR; and PEDro: the Physiotherapy Evidence Database.

A concept framework approach was adopted to design the search strategy around the eligibility criteria for each database (Table 1). Search strings for all included databases are available in S1 File.

### Study selection and screening

In conjunction with the faculty librarian at the host institution of the lead author (RC), a pilot search strategy was developed and tested. Subsequently, appropriate subject headings and keywords were identified for the final search strategy and adapted for each database. All pertinent records identified in the search were collated into Endnote X9.3.3 (Clarivate Analytics, PA, USA) and citation details were imported into the Covidence reference management system (Covidence; Covidence Melbourne, Australia). Duplicates were removed and titles and abstracts were screened by two independent reviewers (RC & CBW) for assessment against the inclusion and exclusion criteria previously outlined. Potentially relevant papers were retrieved and the full text assessed in detail against the inclusion and exclusion criteria by pairs of independent reviewers from the authorship team (ONB, MP, HDB & BDG). Just one disagreement arose between the reviewers at this stage of the selection process which was adjudicated by an additional authorship team member (RS). The appendices of included papers were hand searched for additional relevant studies. This resulted in the addition of a further nine studies.

### Data charting, collection and extraction

Data charting, collection, and extraction for this scoping review followed a systematic and transparent process to ensure comprehensive capture of relevant information. A standardized data extraction form based on the JBI Manual [34] (Appendix 10.1) was used by all members of the research team who completed data extraction. The form was amended to detail key information from eligible studies including: study characteristics (author, year, country of origin), characteristics of instruments (name, purpose, risk factor measured), study design, study population (profession, sex, age), settings, the psychometric performance of instruments where available, and findings related to any relationship between psychosocial factor and injury, where reported. Data extraction was completed by authors RC and CBW and reviewed for accuracy by the remaining members of the authorship team. Disagreements were adjudicated by RS.

## Results

**Fig 1:**
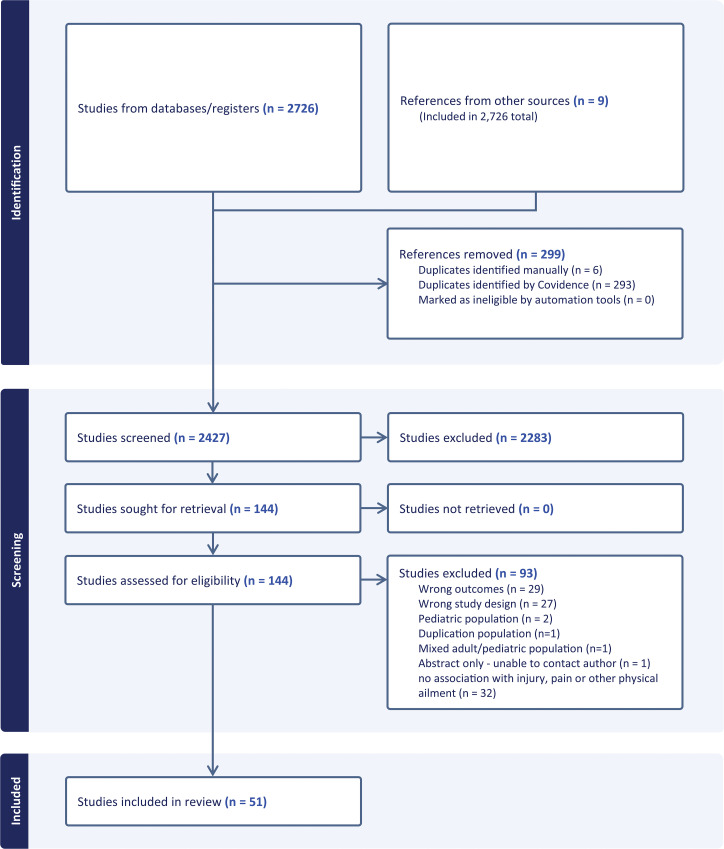
PRISMA Flow diagram of studies screened and included in this review.

Initial searching identified 2726 articles for screening. Following title and abstract review, 144 full texts were assessed against the inclusion and exclusion criteria. Pilots of the title and abstract (n = 270, 10%) and follow-up full text (n = 29, 20%) screening were conducted. An agreement of >85% between reviewers was recorded in both instances. Following full-text review, a total of 51 studies met the inclusion criteria and were included in the scoping review (Fig 1).

**Fig 2:**
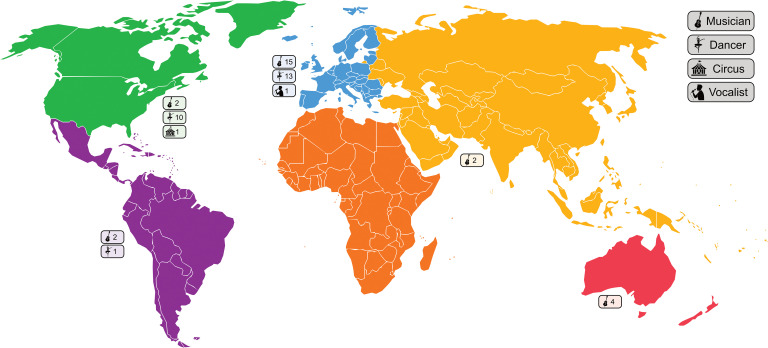
Focus and geographic distribution of studies by continent.

The 51 articles in this review included 25 studies conducted in musicians, 24 in dancers, and a single study each investigating vocalists and circus performers. No studies of theatre performers or other PAs met the criteria for inclusion in this review. The studies were conducted primarily in Europe (n = 29 (56.9%)), and North America (n = 13 (25.5%)), with four (7.8%), three (5.9%) and two (3.9%) studies each from Australia, South America and Asia respectively. Fig 2 outlines the geographical location and cohort focus of the included studies. Studies were published between the years of 1989 and 2024, with 27 (53%) studies published in the ten years since 2015. Just four studies were published prior to 2000, representing a recently expanding body of research in this area.

A total of 7,457 participants were included in this review (male: n = 3,215 (43.1%); female: n = 4,242 (56.9%); unrecorded: n = 11 (0.1%). Participants were drawn from musician (n = 4,505 (60.5%)), dancer (n = 2,680 (35.9%)), vocalist (n = 225 (3.0%)) and circus (n = 47 (0.6%)) populations respectively. Most participants were professional PAs (n = 4,547 (61.0%)), followed by collegiate PAs in full-time programmes of study in their respective arts (n = 1,424 (19.1%)). The balance of participants was drawn from six studies of mixed professional, pre-professional, elite competitive, and collegiate PAs (n = 1,486 (19.9%)). Study characteristics are outlined in S1 Table.

A total of 81 distinct instruments were used by authors to evaluate various psychosocial domains (S2 Table). Additionally, nine bespoke instruments including visual analogue scales and Likert scales were created and used by authors to interrogate various psychosocial criteria of interest. The majority of instruments were just used once across the included studies (n = 68). A number of established instruments were used in multiple studies with the most popular including the K-MPAI [30], and various subscales of the Short-Form Health Survey (SF36) [36], which were both used in five studies. The State-Trait Anxiety Inventory (STAI) [37] was used in four studies and the HADS [32], Profile of Moods State (POMS) [38] questionnaire, and the Rosenberg Self-Esteem Scale [39] were all used in three studies.

Of the 81 instruments used in these studies, the psychometric properties of just 19 were reported in the cohort under investigation. This included 13 instruments used in dance cohorts and six in musicians, one of which has also evaluated in vocalists (S2 Table). Typically, acceptable levels of internal consistency (test-retest reliability) were established for the use of these instruments in the pertinent cohort (S2 Table). A further two instruments; the Dancer Injury Profile Questionnaire [40] and the University of North Texas Musician Health Survey [41] were designed specifically for dancers and musicians respectively. However psychometric analysis of these instruments was unavailable in the literature. Finally, an abbreviated version of the First Multidimensional Perfectionism Questionnaire, [42], was found to be reliable in a cohort of elite opera singers. The reliability and/or validity of most instruments used in the included studies may have been established in other cohorts including athletes, patient groups or the general public, but not specifically in the cohort of PAs under consideration.

A total of 45 different psychosocial factors were explored (Table 2), with many studies assessing multiple factors. There were also many outcome measures which assessed more than one psychosocial factor concurrently (e.g., Depression Anxiety Stress Scale). Table 2 outlines the number of instances each factor was included in the studies in this review. The most investigated psychosocial factors in the reviewed studies were anxiety (general (n = 17, 33%) and performance (n = 11, 22%)); stress (general (n = 10, 20%), and workplace (n = 6, 12%)); depression (n = 9, 18%), and perfectionism (n = 5, 10%). These factors were consistently reported in studies of dancers and musicians, with stress presenting as an issue in the single studies involving vocalists and circus performers. Of all studies included in this review, just four studies – all in dancers – reported no relationship between the psychosocial factor investigated, and injury in participants [43–46]. Details of associations/correlations reported between psychosocial factor and injury are detailed in S1 Table.

**Table 2:** Psychosocial factors investigated in reviewed studies.

Adaptability: N = 1 (2%)	Coping: N = 4 (8%)	Holistic psychological wellness: N = 4 (8%)	Multi-dimensional strain: N = 1 (2%)	Self-esteem: N = 3 (6%)
Alcohol addiction: N = 1 (2%)	Core self-evaluation: N = 1 (2%)	Hypervigilance: N = 1 (2%)	Mental fatigue: N = 1 (2%)	Self-regulation/ efficacy: N = 2 (4%)
Anxiety: N = 17 (33%)	Depersonalisation N = 1 (2%)	Inter-personal sensitivity: N = 2 (4%)	Pain-related anxiety: N = 2 (4%)	Sleep disturbance: N = 2 (4%)
Body image: N = 1 (2%)	Depression: N = 9 (18%)	Job satisfaction: N = 1 (2%)	Passion: N = 1 (2%)	Sleep quality: N = 4 (8%)
Burnout: N = 2 (4%)	Disordered eating: N = 4 (8%)	Workplace stress: N = 6 (12%)	Passion for dance: N = 1 (2%)	Social phobia/ anxiety. N = 4 (8%)
Catastrophising: N = 2 (4%)	Dissociation: N = 2 (4%)	Legacy of trauma: N = 1 (2%)	Perfectionism: N = 5 (10%)	Social support: N = 4 (8%)
Childhood adversity: N = 1 (2%)	Emotional regulation: N = 4 (8%)	Life stressors: N = 2 (4%)	Performance anxiety: N = 11 (22%)	Stress: N = 10 (20%)
Competence and control: N = 1 (2%)	Fear-avoidance behaviours: N = 1 (2%)	Mental disorder: N = 3 (6%)	Personality traits: N = 2 (4%)	Temperament (neuroticism): N = 1 (2%)
Competition anxiety: N = 1 (2%)	Fear-avoidance beliefs: N = 2 (4%)	Mood N = 4 (8%)	Resilience: N = 1 (2%)	Tension: N = 1 (2%)

**Note:** Percentages of each factor presented as a proportion of studies included in this review (n = 51).

## Discussion

This scoping review has collated a comprehensive list of the instruments used to evaluate relationships between various psychosocial factors and the risk of injury in non-recreational adult PAs. The extensive number of studies that met the inclusion criteria for this review underscore the central and consequential role that psychosocial factors such as stress, anxiety, depression and others play in the physical performance and overall wellbeing of dancers and musicians in particular. Further studies are required to establish the extent of this relationship in vocalists and circus performers. This scoping review also furthers the hypothesis that PAs from diverse genres have much in common regarding the psychosocial factors that influence and potentially mitigate injury risk.

While this review has comprehensively illustrated the relationship between psychosocial factors and injury in dancers and musicians, there exists a broad swathe of literature highlighting the existence of these factors in vocalists. The presence of performance anxiety [47], neuroticism [48] occupational stress and perfectionism [28] have all been identified in the literature. Many of these studies did not explore a relationship between these factors and injury and thus were not included in this review. Similarly in professional circus performers, levels of depression, anxiety, stress and overall mental health have been found to be worse than in the general population, but mediated by comparatively higher resilience [49]. Similar findings have also been reported in a cohort of 92 circus student-artists [50]. Qualitatively, the link between adverse psychosocial factors and injury has also been reported in numerous studies of PAs across the various professions. In a cohort of professional orchestral musicians, participants identified a direct relationship between injury and factors including performance, workplace and relationship stress [51]. Qualitative research in elite dance has identified how the drive for perfection and the pressures of competition and oppressive power dynamics may push dancers into pain and injury. This experience is frequently normalised due to a subculture of injury perseverance and concealment in the dance world [52]. Similarly, vocalists have self-reported perceived links between performance anxiety, family pressures, depression and a range of somatic problems [53].

Therefore, it is critically important to design appropriate screening protocols to identify and mitigate the presence of key psychosocial drivers of injury in these PAs. As mentioned, a disproportionate focus on screening of physical traits persists despite the ample evidence of the importance of psychosocial factors. Examples include protocols that solely consider phonatory agility, strength, and stamina in vocalists [54] or focus on range of movement (ROM), hypermobility or balance in dancers [55]. In some cases, efforts have been made to consider a small number of psychosocial factors, such as an evaluation of coping in a ballet dance cohort, but once again the vast majority of the protocol referred to physical elements such as flexibility or balance [56]. Given the array of psychosocial issues identified in this review, and their association with injury risk, a more comprehensive approach is required.

The novel “Dancers, Instrumentalists, Vocalists, and Actors” (DIVA) [29] screening protocol considers a more diverse array of injury risk factors including a checklist of psychosocial factors, and the KMPA-I questionnaire as well as general health, activity details, orthopaedic and vocal/audiology items. This is a welcome development, as it may be used and adapted by PAs from a variety of backgrounds. It may, however, inadequately address important factors such as perfectionism, burnout, coping and other factors which were identified in this review. Work by Rousseau and colleagues in the development of a multi-dimensional, risk-based injury screening model for musicians is encouraging [57]. This evidence-based, stakeholder-informed project considered the impact of approximately 15 different psychosocial factors in addition to potential sources of risk including individual characteristics, biomechanics, posture, life habits, workload and physical condition. In doing so, a truly comprehensive evaluation of the holistic risk and vulnerability profile of the musician is possible. It is equally important however to ensure that appropriate instruments are used to evaluate the presence of the psychosocial factor in question.

In this review, the vast majority of instruments used by authors had not been psychometrically tested in the cohort of interest. Assumptions made regarding the transferability of instruments designed for use in patients, athletes or the general public have not been confirmed in all cases. It is important that appropriate instruments are employed to assess the potential impact of common factors such as anxiety, stress, depression and perfectionism in PA populations. There are plentiful instruments identified in this review that have been designed specifically for various types of PAs, such as the KMPA-I which is appropriate for evaluating performance anxiety in musicians [30] and vocalists [31], and the HADS [32] which has been found to be appropriate for evaluating indicators of anxiety and depression in musicians.

Additionally, there are a host of other instruments not discovered in our search that are appropriate for specific performing arts cohorts including the Scale of Coping with Pain for Dancers (COPAIN-Dancer) [58] and the Mazzarolo Music Performance Anxiety Scale (M-MPAS) [59]. It is encouraging to see an instrument devoted to the evaluation of coping, a previously under addressed and important risk factor in PAs. Similar instruments in other key factors such as perfectionism would be welcomed. These instruments have been developed relatively recently and may not have been available to the authors of many studies included in this review. It may also indicate an encouraging move towards the development of more PA-specific tools. Similar bespoke instruments for vocalists and circus performers are lacking. However, given the shared experiences of PAs from disparate genres, research should explore if these instruments are appropriate, or can be adapted, for use in these under-served groups.

Additional considerations relate to the feasibility of using such instruments in the performing arts setting. The instruments identified in this review were largely self-report and varied in length from a brief 5-item scale (Anxiety and Depression Detector) to the 324-item Adult Personality Inventory (S2 Table). There is therefore a need for pragmatism when choosing appropriate screening instrument, balancing the burden of administering the tool, the resources available to the person/team conducting the screening, and the utility and robustness of the selected instrument.

### Limitations

Although the methodological quality of included studies is beyond the scope of this review, many of the studies were cross-sectional, retrospective studies and therefore of lower methodological quality. The exclusion of non-English language studies may have led to the omission of information relevant to this review. The small number of studies in vocalists, circus performers and other groups such as theatre actors may limit the generalizability of findings. Finally, the review mostly draws from studies conducted in Europe and North America. It may therefore reflect the culture and practice of these locations, which may not necessarily be replicated in other countries. Further research in diverse settings internationally would add greatly to the literature in this area.

## Conclusion

Psychosocial issues are commonplace and associated with pain, injury and other physical problems in non-recreational PAs. A plethora of instruments have been used to evaluate this relationship, many of which have not been established as valid and/or reliable in the population of interest. In addition to the evaluation of physical risk factors for injury, screening protocols should commit to comprehensive evaluation of a diverse array of potential psychosocial factors and measure these using appropriate instruments. Importantly, there is a shared experience of psychosocial problems in PAs which transcend profession. Future research should focus on the development and/or validation of instruments that may be used across the entire PA community. Particular emphasis should centre on instruments to investigate the presence of anxiety, stress, depression, perfectionism and coping in PAs. Cross-cultural, mixed discipline focus groups and Delphi studies are recommended as a robust path to achieve these objectives.

## Supporting information

S1 File Search strings for included databases. (DOCX)

S2 File Preferred Reporting Items for Systematic reviews and Meta-Analyses extension for Scoping Reviews (PRISMA-ScR) Checklist. (DOCX)

S1 Table Details of included studies. (DOCX)

S2 Table Details of instruments identified in scoping review. (DOCX)

## References

[1] Hays KF. Performance Psychology with Performing Artists. Oxford Research Encyclopedia of Psychology. Oxford University Press. 2017. doi: 10.1093/acrefore/9780190236557.013.191

[2] Currey J, Sheng D, Neph Speciale A, Cinquini C, Cuza J, Waite BL. Performing Arts Medicine. Phys Med Rehabil Clin N Am. 2020;31(4):609–32. doi: 10.1016/j.pmr.2020.08.001 32981582

[3] Vassallo AJ, Trevor BL, Mota L, Pappas E, Hiller CE. Injury rates and characteristics in recreational, elite student and professional dancers: A systematic review. J Sports Sci. 2019;37(10):1113–22. doi: 10.1080/02640414.2018.1544538 30481111

[4] Bronner S, McBride C, Gill A. Musculoskeletal injuries in professional modern dancers: a prospective cohort study of 15 years. J Sports Sci. 2018;36(16):1880–8. doi: 10.1080/02640414.2018.1423860 29320307

[5] Jeffries AC, Wallace L, Coutts AJ, Cohen AM, McCall A, Impellizzeri FM. Injury, Illness, and Training Load in a Professional Contemporary Dance Company: A Prospective Study. J Athl Train. 2020;55(9):967–76. doi: 10.4085/1062-6050-477-19 32818965 PMC7534944

[6] Jacobs CL, Cassidy JD, Côté P, Boyle E, Ramel E, Ammendolia C, et al. Musculoskeletal Injury in Professional Dancers: Prevalence and Associated Factors: An International Cross-Sectional Study. Clin J Sport Med. 2017;27(2):153–60. doi: 10.1097/JSM.0000000000000314 26889817

[7] Smith TO, Davies L, de Medici A, Hakim A, Haddad F, Macgregor A. Prevalence and profile of musculoskeletal injuries in ballet dancers: A systematic review and meta-analysis. Phys Ther Sport. 2016;19:50–6. doi: 10.1016/j.ptsp.2015.12.007 27080110

[8] Chan C, Driscoll T, Ackermann BJ. Effect of a musicians’ exercise intervention on performance-related musculoskeletal disorders. Med Probl Perform Art. 2014;29(4):181–8. doi: 10.21091/mppa.2014.4038 25433253

[9] Kok LM, Vlieland TPMV, Fiocco M, Nelissen RGHH. A comparative study on the prevalence of musculoskeletal complaints among musicians and non-musicians. BMC Musculoskelet Disord. 2013;14:9. doi: 10.1186/1471-2474-14-9 23289849 PMC3554565

[10] Rodríguez-Gude C, Taboada-Iglesias Y, Pino-Juste M. Musculoskeletal pain in musicians: prevalence and risk factors - a systematic review. Int J Occup Saf Ergon. 2023;29(2):883–901. doi: 10.1080/10803548.2022.2086742 35678565

[11] Mehta DD, Zañartu M, Feng SW, Cheyne HA 2nd, Hillman RE. Mobile voice health monitoring using a wearable accelerometer sensor and a smartphone platform. IEEE Trans Biomed Eng. 2012;59(11):3090–6. doi: 10.1109/TBME.2012.2207896 22875236 PMC3539821

[12] Pestana PM, Vaz-Freitas S, Manso MC. Prevalence of Voice Disorders in Singers: Systematic Review and Meta-Analysis. J Voice. 2017;31(6):722–7. doi: 10.1016/j.jvoice.2017.02.010 28342677

[13] Kridgen S, Hillman RE, Stadelman-Cohen T, Zeitels S, Burns JA, Hron T, et al. Patient-Reported Factors Associated with the Onset of Hyperfunctional Voice Disorders. Ann Otol Rhinol Laryngol. 2021;130(4):389–94. doi: 10.1177/0003489420956379 32909443 PMC7940573

[14] Bird HA. Overuse syndrome in musicians. Clin Rheumatol. 2013;32(4):475–9. doi: 10.1007/s10067-013-2198-2 23392828

[15] Phyland D. The Measurement and Effects of Vocal Load in Singing Performance. How Much Singing Can a Singer Sing if a Singer Can Sing Songs?. Perspect ASHA SIGs. 2017;2(3):79–88. doi: 10.1044/persp2.sig3.79

[16] Fuller M, Moyle GM, Hunt AP, Minett GM. Ballet and Contemporary Dance Injuries When Transitioning to Full-Time Training or Professional Level Dance: A Systematic Review. J Dance Med Sci. 2019;23(3):112–25. doi: 10.12678/1089-313X.23.3.112 31500693

[17] Rietveld ABMB. Dancers’ and musicians’ injuries. Clin Rheumatol. 2013;32(4):425–34. doi: 10.1007/s10067-013-2184-8 23572035

[18] Knapik JJ, Jones SB, Darakjy S, Hauret KG, Nevin R, Grier T, et al. Injuries and injury risk factors among members of the United States Army Band. Am J Ind Med. 2007;50(12):951–61. doi: 10.1002/ajim.20532 17979136

[19] Baadjou VAE, Roussel NA, Verbunt JAMCF, Smeets RJEM, de Bie RA. Systematic review: risk factors for musculoskeletal disorders in musicians. Occup Med (Lond). 2016;66(8):614–22. doi: 10.1093/occmed/kqw052 27138935

[20] Kenny SJ, Whittaker JL, Emery CA. Risk factors for musculoskeletal injury in preprofessional dancers: a systematic review. Br J Sports Med. 2016;50(16):997–1003. doi: 10.1136/bjsports-2015-095121 26626269

[21] Backiev L, Bastepe-Gray S, Mueller D, Watson MD, Chiang C-C, Emam M, et al. Updates in Performing Arts Medicine: A Clinical Overview for Instrumental Musicians and Dancers. Curr Phys Med Rehabil Rep. 2024;12(2):223–33. doi: 10.1007/s40141-024-00450-w

[22] Jacukowicz A. Psychosocial work aspects, stress and musculoskeletal pain among musicians. A systematic review in search of correlates and predictors of playing-related pain. Work. 2016;54(3):657–68. doi: 10.3233/WOR-162323 27315412

[23] Donohue B, Gavrilova Y, Galante M, Burnstein B, Aubertin P, Gavrilova E, et al. Empirical development of a screening method for mental, social, and physical wellness in amateur and professional circus artists. Psychology of Aesthetics, Creativity, and the Arts. 2020;14(3):313–24. doi: 10.1037/aca0000199

[24] Shrier I, Raglin JS, Levitan EB, Mittleman MA, Steele RJ, Powell J. Procedures for assessing psychological predictors of injuries in circus artists: a pilot prospective study. BMC Med Res Methodol. 2014;14:77. doi: 10.1186/1471-2288-14-77 24920527 PMC4064279

[25] Mainwaring LM, Finney C. Psychological Risk Factors and Outcomes of Dance Injury: A Systematic Review. J Dance Med Sci. 2017;21(3):87–96. doi: 10.12678/1089-313X.21.3.87 28871899

[26] Wainwright SP, Williams C, Turner BS. Fractured identities: injury and the balletic body. Health (London). 2005;9(1):49–66. doi: 10.1177/1363459305048097 15576424

[27] Toner J, Jones L, Moran A. Bodily crises in skilled performance: Considering the need for artistic habits. Performance Enhancement & Health. 2016;4(1–2):50–7. doi: 10.1016/j.peh.2015.10.001

[28] Pecen E, Collins DJ, MacNamara Á. “It’s Your Problem. Deal with It.” Performers’ Experiences of Psychological Challenges in Music. Front Psychol. 2018;8:2374. doi: 10.3389/fpsyg.2017.02374 29422878 PMC5788962

[29] Ackermann BJ, Guptill C, Miller C, Dick R, McCrary JM. Assessing Performing Artists in Medical and Health Practice - The Dancers, Instrumentalists, Vocalists, and Actors Screening Protocol. Curr Sports Med Rep. 2022;21(12):460–2. doi: 10.1249/JSR.0000000000001022 36508603

[30] Kenny DT. The Kenny music performance anxiety inventory (K-MPAI): Scale construction, cross-cultural validation, theoretical underpinnings, and diagnostic and therapeutic utility. Front Psychol. 2023;14:1143359. doi: 10.3389/fpsyg.2023.1143359 37325731 PMC10262052

[31] Kenny DT, Davis P, Oates J. Music performance anxiety and occupational stress amongst opera chorus artists and their relationship with state and trait anxiety and perfectionism. J Anxiety Disord. 2004;18(6):757–77. doi: 10.1016/j.janxdis.2003.09.004 15474851

[32] Zigmond AS, Snaith RP. The hospital anxiety and depression scale. Acta Psychiatr Scand. 1983;67(6):361–70. doi: 10.1111/j.1600-0447.1983.tb09716.x 6880820

[33] Munn Z, Peters MDJ, Stern C, Tufanaru C, McArthur A, Aromataris E. Systematic review or scoping review? Guidance for authors when choosing between a systematic or scoping review approach. BMC Med Res Methodol. 2018;18(1):143. doi: 10.1186/s12874-018-0611-x 30453902 PMC6245623

[34] Aromataris E, Munn Z. JBI manual for evidence synthesis. Adelaide: JBI. 2020.

[35] Tricco AC, Lillie E, Zarin W, O’Brien KK, Colquhoun H, Levac D, et al. PRISMA Extension for Scoping Reviews (PRISMA-ScR): Checklist and Explanation. Ann Intern Med. 2018;169(7):467–73. doi: 10.7326/M18-0850 30178033

[36] Ware JE Jr, Sherbourne CD. The MOS 36-item short-form health survey (SF-36). I. Conceptual framework and item selection. Med Care. 1992;30(6):473–83. doi: 10.1097/00005650-199206000-00002 1593914

[37] Spielberger CD, Gonzalez-Reigosa F, Martinez-Urrutia A, Natalicio LFS, Natalicio DS. Interam J Psychol. 2017;5(3).

[38] McNair DM, Lorr M, Droppleman LF. Manual profile of mood states. San Diego: Educational and Industrial Testing Service. 1971.

[39] Rosenberg M. Society and the adolescent self-image. Princeton, New Jersey, United States: Princeton University Press. 1965.

[40] Rip B, Fortin S, Vallerand RJ. The Relationship between Passion and Injury in Dance Students. Journal of Dance Medicine & Science. 2006;10(1–2):14–20. doi: 10.1177/1089313x06010001-205

[41] Pak CH, Chesky K. Prevalence of Hand, Finger, and Wrist Musculoskeletal Problems in Keyboard Instrumentalists: The University of North Texas Musician Health Survey. Medical Problems of Performing Artists. 2001;16(1):17–23. doi: 10.21091/mppa.2001.1004

[42] Frost RO, Marten P, Lahart C, Rosenblate R. The dimensions of perfectionism. Cogn Ther Res. 1990;14(5):449–68. doi: 10.1007/bf01172967

[43] Beriet GC, Kiebzak GM, Dandar A, Wooten C, Box JH, Anderson RB, et al. Prospective Analysis of Body Composition and SF36 Profiles in Professional Dancers over a 7-Month Season: Is There a Correlation to Injury?. Journal of Dance Medicine & Science. 2002;6(2):54–61. doi: 10.1177/1089313x0200600205

[44] Byhring S, Bø K. Musculoskeletal injuries in the Norwegian National Ballet: a prospective cohort study. Scand J Med Sci Sports. 2002;12(6):365–70. doi: 10.1034/j.1600-0838.2002.01262.x 12453164

[45] Hilgenberg-Sydney PB, Wilhelm JM, Pimentel G, Petterle R, Bonotto D. Prevalence of Temporomandibular Disorders and Anxiety State Levels in Ballet Dancers A Cross-Sectional Study. J Dance Med Sci. 2020;24(2):88–92. doi: 10.12678/1089-313X.24.2.88 32456763

[46] Nordin-Bates SM, Walker IJ, Baker J, Garner J, Hardy C, Irvine S, et al. Injury, Imagery, and Self-esteem in Dance. Journal of Dance Medicine & Science. 2011;15(2):76–85. doi: 10.1177/1089313x1101500204 21703096

[47] Henshaw A, Collyer S. Under Pressure: Reports of Performance Anxiety Across Multiple Singing Genres. SING. 2022;78(5):583–90. doi: 10.53830/jeta7812

[48] Sielska-Badurek EM, Sobol M, Okulicz-Kozaryn K, Gołda P, Cielecka A. Personality traits in singers performing various music styles and with different singing status. Int J Occup Med Environ Health. 2023;36(4):541–50. doi: 10.13075/ijomeh.1896.02099 37750428 PMC10694793

[49] van Rens FECA, Heritage B. Mental health of circus artists: Psychological resilience, circus factors, and demographics predict depression, anxiety, stress, and flourishing. Psychology of Sport and Exercise. 2021;53:101850. doi: 10.1016/j.psychsport.2020.101850

[50] Decker A, Richard V, Cairney J, Jefferies P, Houser N, Aubertin P, et al. Assessment of Professional Circus Students’ Psychological Characteristics at Four Strategic Timepoints over the Scholastic Year: A Longitudinal Study Using the Stress Process Model. Med Probl Perform Art. 2022;37(4):249–58. doi: 10.21091/mppa.2022.4036 36455109

[51] Injury and the Orchestral Environment: Part I. The Role of Work Organisation and Psychosocial Factors in Injury Risk. Medical Problems of Performing Artists. 2013;28(4):219–29. doi: 10.21091/mppa.2013.4043 24337034

[52] McEwen K, Young K. Ballet and pain: reflections on a risk-dance culture. Qualitative Research in Sport, Exercise and Health. 2011;3(2):152–73. doi: 10.1080/2159676x.2011.572181

[53] Sandgren M. Voice, Soma, and Psyche: A Qualitative and Quantitative Study of Opera Singers. Medical Problems of Performing Artists. 2002;17(1):11–21. doi: 10.21091/mppa.2002.1003

[54] Stadelman-Cohen T, Burns J, Zeitels S, Hillman R. Team Management of Voice Disorders in Singers. Leader. 2009;14(15):12–5. doi: 10.1044/leader.ftr1.14152009.12

[55] Armstrong R, Relph N. Screening Tools as a Predictor of Injury in Dance: Systematic Literature Review and Meta-analysis. Sports Med Open. 2018;4(1):33. doi: 10.1186/s40798-018-0146-z 30022294 PMC6051954

[56] Critchley ML, Bonfield S, Ferber R, Pasanen K, Kenny SJ. Relationships Between Common Preseason Screening Measures and Dance-Related Injuries in Preprofessional Ballet Dancers. J Orthop Sports Phys Ther. 2023;53(11):703–11. doi: 10.2519/jospt.2023.11835 37787614

[57] Rousseau C, Barton G, Garden P, Baltzopoulos V. Development of an injury prevention model for playing-related musculoskeletal disorders in orchestra musicians based on predisposing risk factors. International Journal of Industrial Ergonomics. 2021;81:103026. doi: 10.1016/j.ergon.2020.103026

[58] Becker da Silva AM, Enumo SRF, Carvalho LdF, Machado WdL, Bittencourt IG, Araújo MFd, et al. Scale of Coping with Pain for Dancers (COPAIN-Dancer): Construction and validity evidences. PSICO. 2019;37(1):159–93. doi: 10.18800/psico.201901.006

[59] Mazzarolo I, Schubert E. A Short Performance Anxiety Scale for Musicians. Front Psychol. 2022;12:781262. doi: 10.3389/fpsyg.2021.781262 35153903 PMC8826570

