# Supplementary material for: Psychosocial risk factors for injury in performing artists: A scoping review of screening and assessment instruments
Source: PLoS One. 2026 Feb 24;21(2):e0322971. doi: 10.1371/journal.pone.0322971 (PMC12931760; doi:10.1371/journal.pone.0322971)
Supplement: S2 Table — (DOCX) [file pone.0322971.s004.docx]

| **Instrument Name** | **Instrument Description** | **Primary Outcome Measure (s) Evaluated** | **Frequency of use in identified studies** | **Psychometric Testing in Dancers/Musicians/Vocalists/**  **Circus** | **Psychometric Properties** |
| --- | --- | --- | --- | --- | --- |
| Adult Personality Inventory (API)(1) | 324-item scale assessing normal-range adult personality traits across personal characteristics, interpersonal style, and career factors | Personality traits | 1 | Not reported | N/A |
| Adverse Childhood Experiences (ACE)(2) | 10-item questionnaire asking about experiences of abuse, neglect, and household challenges before the age of 18 | Childhood adversity mediated negative health outcomes in adulthood; e.g. risk of chronic diseases, mental health disorders, and premature mortality. | 1 | Not reported | N/A |
| Alcohol Use Disorders Identification Test (AUDIT)(3) | 10-item questionnaire that screens for hazardous or harmful alcohol consumption | Alcohol addiction | 1 | Not reported | N/A |
| Anxiety and Depression Detector (ADD)(4) | 5-item brief screening tool designed to identify individuals at risk for anxiety and depressive disorders in primary care settings | Anxiety and/or depression | 1 | Not reported | N/A |
| Athletic Coping Skills Inventory (ACSI-28)(5) | A self-report assessment designed to evaluate athletes' psychological coping skills across seven key areas including:  Coping with adversity,  peaking under pressure,  goal setting and mental preparation,  concentration,  freedom from worry  confidence and achievement motivation  coachability | Psychological coping skills | 2 |  |  |
| Athlete Burnout Questionnaire (ABQ)(6) | 15-item self-report tool that assesses burnout in athletes across three dimensions: emotional and physical exhaustion, reduced sense of accomplishment, and devaluation of sport participation | Burnout | 1 | Tested for validity in dancers.(7)  Not evaluated in musicians, vocalists or circus performers. | A confirmatory factor analysis was conducted on the modified 15-item ABQ for dancers and athletes, which did not fit the model fit criteria. |
| Beck Anxiety Inventory (BAI-II)(8) | 21-item self-report questionnaire designed to assess the severity of anxiety symptoms in adults and adolescents. | Anxiety | 2 | Not reported | N/A |
| Beck Depression Inventory II (BDI-II)(9) | 21-item self-report inventory measuring the severity of depression in adolescents and adults | Depression | 1 | Evaluated for internal consistency in dancers.(10)  Not evaluated in musicians, vocalists or circus performers. | The study reported high internal consistency, with Cronbach's alpha coefficients of 0.86 for pretreatment, 0.91 for posttreatment, and 0.91 for follow-up measurements. |
| Bergen Insomnia Scale(11) | 6-item self-report questionnaire designed to assess insomnia symptoms and is rated based on frequency over the past month. | Sleep disturbance | 1 | Not reported | N/A |
| Body Appreciation Scale-2 (BAS-2)(12) | 13-item self-report questionnaire that assess body appreciation and positive body image. | Body image | 1 | Internal consistency tested in recreational pole-dancers.(13)  Not evaluated in musicians, vocalists or circus performers. | High internal consistency was reported with Cronbach’s α values for BAS-2 pre-training and post-training scores were 0.95 and 0.94, respectively |
| Brief COPE(14) | A self-report questionnaire that assesses a range of coping strategies individuals use in response to stress, consisting of 28 items across 14 coping subscales. | Coping with stress | 1 | A modified COPE for ballet dancers was evaluated for reliability (internal consistency), factor structure and construct validity.(15)  Not evaluated in musicians, vocalists or circus performers. | Cronbach's alpha values for different subscales ranged from 0.60 to 0.85, indicating acceptable to good reliability for most coping strategies.  Factor analyses supported the scale's ability to differentiate between coping strategies.  The scale demonstrated good construct validity in identifying coping strategies linked to competitive anxiety and stress among dancers. |
| Cambridge Depersonalisation Scale (CD-9)(16) | 29-item self-administered questionnaire that measures the severity of depersonalisation experiences, such as feeling detached from oneself or observing one's thoughts and actions from outside the body. | Depersonalisation | 1 | Not reported | N/A |
| Children and Adolescent Perfectionism Scale (CAPS)(17) | 22-item self-administered questionnaire measuring perfectionistic tendencies in young people, including aspects such as self-criticism, concern over mistakes, and the pursuit of high standards. | Perfectionism | 1 | Internal consistency tested in competitive Irish dancers.(18)  Not evaluated in musicians, vocalists or circus performers. | CAPS demonstrated satisfactory internal consistency, with Cronbach's alpha coefficients ranging from 0.70 to 0.84 |
| Cohen Perceived Stress Scale(19) | 10-item self-administered questionnaire measuring the degree to which individuals perceive their life as stressful, assessing feelings of unpredictability, uncontrollability, and overload in daily life | Stress | 1 | Internal consistency and construct validity tested in Indian Kathak dancers.(20)  Not evaluated in musicians, vocalists or circus performers. | Internal consistency: Cronbach's alpha coefficients of 0.76 for dancers. The scale's construct validity was also confirmed. |
| Competitive Trait Anxiety Inventory (CTAI)(21) | 15-item self-administered questionnaire that measures an individual's level of trait anxiety in competitive situations, assessing the tendency to experience anxiety in competitive settings. | Competition anxiety | 1 | Internal consistency tested in 20 musicians with focal dystonia.(22)  Not evaluated in dancers, vocalists or circus performers. | Cronbach's alpha values of 0.81 for the "somatic anxiety" subscale and 0.83 for the "self-doubt concern" subscale, indicating satisfactory internal consistency. |
| Copenhagen Burnout Inventory (CBI)(23) | 19-item self-administered questionnaire measuring burnout across three dimensions: personal burnout, work-related burnout, and client-related burnout, assessing physical and psychological exhaustion | Burnout | 1 | Not reported | N/A |
| Coping Inventory for Stressful Situations (CISS)(24) | 48-item self-administered measuring three coping styles—task-oriented, emotion-oriented, and avoidance-oriented—assessing how individuals respond to stress. | Coping with stress | 1 | Internal consistency tested in a cohort of pre-professional and professional dancers.(25)  Not evaluated in musicians, vocalists or circus performers. | Excellent internal consistency with Cronbach's alpha coefficients of 0.84 for task-oriented focus, 0.90 for emotion-oriented focus, and 0.86 for avoidance-oriented coping. |
| Coping Strategies Questionnaire (CSQ)(26) | 50-item self-administered tool measuring cognitive and behavioural coping strategies used to manage pain, including catastrophising, ignoring sensations, reinterpreting pain, and seeking social support. | Coping | 1 | Not reported | N/A |
| Core Self Evaluation Scale (CSE)(27) | 12-item self-administered questionnaire measuring an individual's fundamental self-assessments, including self-esteem, generalized self-efficacy, locus of control, and emotional stability (neuroticism) | Core self-evaluation | 1 | Not reported | N/A |
| Dancer Injury Profile Questionnaire(28) | 21 items  assessing physical injury characteristics as well as coping behaviours in responses  to pain and injury, items assessing health-promoting, as well as health-undermining coping behaviours. | Coping and health promotion behaviours. | 1 | Not reported | N/A |
| Depression Anxiety Stress Scale (DASS)(29) | 42-item self-report instrument designed to measure three related negative emotional states, namely depression, anxiety and stress. | Depression, anxiety and stress. | 1 | Not reported | N/A |
| Difficulties in Emotion Regulation Scale (DERS)(30) | 36-item self-report measure of six facets of emotion regulation, namely Nonacceptance of Emotional Responses, Difficulties in Engaging in Goal-Directed Behaviour, Impulse Control Difficulties, Lack of Emotional Awareness, Limited Access to Emotion Regulation Strategies, and Lack of Emotional Clarity | Emotional regulation | 1 | Not reported | N/A |
| Dissociative Experience Scale—II (DES-II)(31) | 28-item self-administered questionnaire related to experiences of dissociation, such as depersonalization, derealization, amnesia, and absorption. | Dissociation | 1 | Not reported | N/A |
| Eating Disorders Inventory 2 (EDI-2)(32) | 91-items self-administered questionnaire of subscales assessing attitudes, behaviours, and psychological traits related to eating disorders, such as drive for thinness, bulimia, and body dissatisfaction. | Disordered eating. | 1 | Not reported | N/A |
| Eating disorder examination questionnaire (EDE-QS)(33) | 28-items self-administered questionnaire that assess the frequency and severity of eating disorder behaviours and attitudes, such as restraint, eating, shape, and weight concerns. | Disordered eating. | 1 | Not reported | N/A |
| Fear-Avoidance Beliefs Questionnaire (FABQ)(34) | 16-items self-administered questionnaire including two subscales that assess fear-avoidance beliefs related to physical activity and work in individuals with back pain or musculoskeletal disorders. | Fear-avoidance beliefs | 1 | Not reported | N/A |
| Fonseca Anamnestic Questionnaire(35) | 10-item self-administered questionnaire designed to assess symptoms related to temporomandibular disorders (TMD), such as pain, jaw movement, and headaches. | Tension (and other physical factors) related to TMD | 1 | Not reported | N/A |
| Formal Characteristics of  Behaviour–Temperament Inventory (FCB-TI)(36) | 120 yes/no questions, divided into six subscales measuring traits such as: briskness, perseverance, sensory sensitivity, emotional reactivity,  endurance, and activity | Temperament | 1 | Not reported | N/A |
| Freiburg Personality Inventory - Revised (FPI-R)(37) | 138 item self-administered questionnaire assessing various personality traits, such as emotional stability, social behaviour, and self-confidence | Personality | 1 | Not reported | N/A |
| Frost Multidimensional Perfectionism Scale(38) | 35-item question self-report measure with four sub-scales of perfectionism. | Perfectionism | 1 | Not reported | Note: Abridged version of this instrument has been found to have internal reliability in elite vocalists. Cronbach’s alpha = 084.(39) |
| General Health Questionnaire-12(40) | 12-items self-administered questionnaire to assess psychological well-being, focusing on symptoms of anxiety, depression, and social dysfunction | Psychological well-being. | 1 | Not reported | N/A |
| General Self-Efficacy Scale (GSES)(41) | 10-item self-administered questionnaire which measures an individual's belief in their ability to cope with a variety of challenging or stressful situations | Self-efficacy/ coping | 1 | Not reported | N/A |
| Generalised Anxiety Disorder Assessment (GAD-7)(42) | 7-item self-reported instrument that is used to measure or assess the severity of generalised anxiety over the previous two weeks. | Anxiety | 1 | Internal consistency tested in Indian Kathak dancers.(20)  Not evaluated in musicians, vocalists or circus performers. | Cronbach’s α for the internal consistency of the GAD-7 was 0.84 for Kathak dancers |
| Hopkins Symptom Checklist-25 (HSCL-25)(43) | 58-item checklist scored on five underlying symptom dimensions—somatization, obsessive-compulsive, interpersonal sensitivity, anxiety and depression. | Anxiety, depression, interpersonal sensitivity. | 1 | Internal consistency tested in cohort of professional Norwegian musicians.(44)  Not evaluated in dancers, vocalists or circus performers. | Cronbach's α (internal consistency) of 0.93 for the total HSCL-25 score, indicating strong reliability. |
| Hospital Anxiety and Depression Scale (HADS)(45) | 14-item self-assessed tool that measures anxiety and depression levels in individuals, with 7 items for each dimension. | Anxiety and depression | 3 | Internal consistency tested in cohort of professional Danish musicians.(46)  Not evaluated in dancers, vocalists or circus performers. | Cronbach's α (internal consistency) of 0.89 for HADS-Anxiety, and 0.82 for HADS-Depression scores. |
| Job Stress Scale (JSS)(47) | 13 item self-administered scale that measures job stress, focusing on various factors like workload, role conflict, and social support | Job stress | 1 | Not reported | N/A |
| Karasek Model: demand, support and control at work(48) | 49-item self-assessment questionnaire that measures job demands, decision latitude (control), and social support to evaluate workplace stress and its impact on employee well-being. | Occupational stress. | 1 | Not reported | N/A |
| Kenny Music Performance Anxiety Inventory (K-MPAI)(49) | 40-item inventory that assesses an emotion-based theory of anxiety as it applies to anxiety in the context of music performance. | Performance anxiety | 5 | Widely examined in multiple cohorts of musicians.(49)  Additionally evaluated in a cohort of 32 elite operatic chorus artists.(39)  Not evaluated in dancers or circus performers. | K-MPAI consistently demonstrates a stable factorial structure, robust reliability, and strong utility for diagnostic purposes among musicians, across various studies and cultural contexts.(49)  Internal reliability of K-MPAI in vocalists: Cronbach’s alpha = 0.944(39) |
| Kessler Psychological Distress Scale(50) | 10-item self-report questionnaire designed to measure the level of psychological distress based on anxiety and depressive symptoms experienced over the past 30 days. | Anxiety and Depression | 1 | Not reported | N/A |
| Life Experiences Survey (LES)(51) | 57-item self-report questionnaire which assess various life events and their impact, categorising them as either positive or negative stressors | Life stressors | 1 | Not reported | N/A |
| Mental Health Inventory-5(52) | 5-item self-administered questionnaire assessing psychological well-being and distress, including aspects like anxiety, depression, positive affect, and general well-being | Psychological well-being | 1 | Not reported | N/A |
| Mental Health Test (MHT)(53) | 17-item self-administered scale that assesses various aspects of mental health, including symptoms related to mood, anxiety, and general psychological well-being. | Mental health | 1 | Not reported | N/A |
| Minnesota Satisfaction Questionnaire-Short (MSQ)(54) | 100-item self-administered questionnaire assessing 20 different facets of job satisfaction. | Job satisfaction | 1 | Not reported | N/A |
| Modified Fatigue Impact Scale (MFIS)(55) | 21-item self-administered questionnaire assessing fatigue on a person's physical, cognitive, and psychosocial functioning. | Multifactorial fatigue | 1 | Not reported | N/A |
| Musculoskeletal Pain Intensity and Interference Questionnaire (MPIIIQ)(56) | 9-item self-administered questionnaire that assesses the intensity of musculoskeletal pain and its impact on daily activities, mood, and performance in professional musicians. | Pain-related mood, performance. | 1 | Evaluated in professional musicians.(56)  Not evaluated in dancers, vocalists or circus performers. | Internal consistency: Cronbach's alpha of 0.91 for pain intensity and pain interference subscales.  Test-Retest Reliability: intraclass correlation coefficients ranging from 0.78 to 0.82 and moderate to substantial reliability for pain interference items (coefficients ranging from 0.56 to 0.76).  Factor Structure: Exploratory factor analysis revealed a stable two-factor structure, accounting for 71.3% of the variance, identifying distinct pain intensity and pain interference components. |
| National Athletic Trainers Association Mental Health Screening Tool(57) | 9-item self-report measure that asks for yes/no responses on a range of mental health topics. | General mental health. | 1 | Not reported | N/A |
| National Institute for Occupational Safety and Health (NIOSH) Generic Job Stress Survey(58) | Measures 13  different job stressors as well as a host of measures of individual distress and modifiers of the  stress response | Job stress | 1 | Not reported | N/A |
| Neuroticism Ekstraversion Openness–Five Factor Inventory (NEO-FFI)(59) | 60 self-reporting statements rated on a 5-point scale measuring: neuroticism, extraversion, openness to experience, agreeableness, and conscientiousness | Personality and adaptation capability | 1 | Not reported | N/A |
| Occupational Environmental Stress (OES) scale(60) | 48 item scale which assess different aspects of stress in the workplace, focusing on both environmental and organizational factors that contribute to job stress. | Occupational stress | 1 | Not reported | N/A |
| Örebro Musculoskeletal Pain Screening Questionnaire (OMPSQ)(61) | 25-items self-completed tool assessing psychosocial and physical factors related to musculoskeletal pain, including pain intensity, disability, and the risk of developing chronic pain or disability. | Biopsychosocial factors associated with pain. | 1 | Not reported | N/A |
| Pain Anxiety Symptom Scale-Short (PASS-20)(62) | 20-item self-completed questionnaire that assesses the severity of pain-related anxiety by measuring symptoms such as fear, avoidance, and hypervigilance in response to pain. | Fear avoidance, pain hypervigilance. | 1 | Not reported | N/A |
| Pain Catastrophizing Scale (PCS)(63) | 13-item self-report measure of catastrophising in the context of actual or anticipated pain | Catastrophising | 1 | Not reported | N/A |
| Passion for Dance Scale (PDS)(28) | Composed of two six-item subscales  assessing harmonious and obsessive  passion for dance, as well as four  passion criterion items measuring  whether dance could be considered  a “passion” for each participant | Passion for dance | 1 | Internal consistency tested in a cohort of mixed-genre dancers.(28)  Not evaluated in musicians, vocalists or circus performers. | Internal consistency  indices of .78 and .84 were obtained  for the harmonious and obsessive  passion subscales, respectively. |
| Passion Scale (PS)(64) | Composed of two seven-item subscales  assessing harmonious and obsessive  passion for a given activity. | Passion | 1 | Not reported | N/A |
| Patient Health Questionnaire-9 (PHQ-9)(65) | 9-item self-reported tool that assess the severity of depressive symptoms over the past two weeks, focusing on mood, interest, energy, and related aspects of depression | Mood, depression. | 2 | Not reported | N/A |
| Perceived Events Scale (PES)(66) | 207 questions pertaining to events covering a wide range of life domains. For each item, the participants indicated whether the event had occurred in the past 6 months. | Positive and negative life events. | 2 | Not reported | N/A |
| Perfectionism Inventory (Dance version)(67) | Includes seven scales assessing various domains of perfectionism.  The first three subscales constitute the factor  conscientious perfectionism, while the latter four address self-evaluative perfectionism. | Perfectionism | 1 | Internal consistency evaluated in cohort of contemporary and ballet dancers.(67)  Not evaluated in musicians, vocalists or circus performers. | Internal  reliability per subscale (Cronbach’s alpha values) ranging from  0.74 to 0.89 |
| Performance Anxiety Questionnaire (PAQ)(68) | Measures both  cognitive anxiety (10 items) and physiological anxiety  (10 items) related to musical performance in three different  contexts (solo performance, chamber music performance  and orchestral performance). | Performance anxiety. | 1 | Internal consistency evaluated in cohort of music students.(69)  Not evaluated in dancers, vocalists or circus performers. | Internal consistency of Cronbach’s α = .90 and correlated at  r = .60, p < .001 with the negative affect scale. |
| Personal Resources Questionnaire (PRQ)(70) | A 2-part measure of the multidimensional characteristics of social support. Part1 pertains to resources and supports, and Part 2 relates to self-help. | Situational and perceived social support | 1 | Not reported | N/A |
| Personal Strain Questionnaire (PSQ)(60) | 40-item self report instrument including four areas namely: Vocational Strain, Psychological Strain, Interpersonal Strain , Physical Strain. | Multidimensional strain | 1 | Not reported | N/A |
| Pittsburgh Sleep Quality Index (PSQI)(71) | A self-report questionnaire that assesses sleep quality over a 1-month time interval. The measure consists of 19 individual items, creating 7 components that produce one global score | Sleep quality | 2 | Not reported | N/A |
| Positive and Negative Affect Scale (PANAS)(72) | This brief scale is comprised of 20 items, with 10 items measuring positive affect (e.g., excited, inspired) and 10 items measuring negative affect (e.g., upset, afraid). | Mood/emotion | 1 | Not reported | N/A |
| PRIME-MD Patient Health Questionnaire (PRIME-MD PHQ)(65) | A self-administered 1-page questionnaire consisting of 26 yes/no questions about the presence of symptoms and signs of common mental disorders during the past month | Mental disorder | 2 | Not reported | N/A |
| Profile of Mood States (POMS)(73) | POMS measures six different dimensions of mood swings over a period of time. These include: Tension or Anxiety, Anger or Hostility, Vigor or Activity, Fatigue or Inertia, Depression or Dejection, Confusion or Bewilderment. | Mood | 3 | Not reported | N/A |
| Questionnaire for Competence and Control Orientations (QCC)(74) | QCC investigates features such as self-concept of abilities, internal control  orientation, others control orientation, chance control  orientation | Competence and control | 1 | Not reported | N/A |
| Recovery-Stress Questionnaire for Athletes (RESTQ-Sport)(75) | 52-item subjective questionnaire sensitive to the stress and recovery incurred by sports and general lifestyles | Stress and recovery | 2 | Dance version of the REST-Q has been developed and evaluated.(76)  Not evaluated in musicians, vocalists or circus performers. | Factor Structure: The RESTQ-Dance comprises 63 items organized into three factors:  Internal consistency:  General Stress: 26 items (Cronbach's α = 0.92)  Recovery: 27 items (Cronbach's α = 0.91)  Specific Stress: 10 items (Cronbach's α = 0.79)  Internal Consistency: Cronbach's alpha values for the factors indicate good internal consistency, with values ranging from 0.79 to 0.92. |
| Resilience Scale for Adults(77) | 25-item self-report scale designed to assess the level of resilience in adults. Includes dimensions of personal resilience, such as personal competence, acceptance of self and life, and social competence | Resilience and coping | 1 | Not reported | N/A |
| Rosenberg Self-Esteem Scale(78) | 10-item self-report scale that measures global self-worth by measuring both positive and negative feelings about the self | Self-esteem | 3 | Not reported | N/A |
| Short-form Self-Regulation Questionnaire(79) | 31-item instrument designed to assess their capacity  for self-regulation; that is, the ability to plan, guide, and monitor  behaviours in the face of changing circumstances | Self-regulation | 1 | Not reported | N/A |
| Short Symptom Check List (SCL-10)(80) | 10 questions scored from 0 to 4,  and all are averaged into a global score, in which higher scores present higher severity of  symptoms. A cut-off score of 1.85 indicates symptoms of mental health issues. | General health issues including mental health. | 1 | Not reported | N/A |
| Short-Form Health Survey SF36(81) | 36-item questionnaire that assesses health-related quality of life across eight domains, including physical functioning, pain, emotional well-being, and social functioning | Health related quality of life | 5 | Not reported | N/A |
| Sick, Control, One stone, Fat, Food (SCOFF) questionnaire(82) | A brief, 5-item measure that has been used to screen for anorexia nervosa and bulimia nervosa | Disordered eating | 2 | Not reported | N/A |
| Social Phobia Inventory (SPIN)(83) | 17-item self-rating scale for social anxiety disorder (social phobia). The scale is rated over the past week and includes items assessing each of the symptom domains of social anxiety disorder (fear, avoidance, and physiologic arousal) | Social phobia/ anxiety. | 1 | Not reported | N/A |
| Social Support Appraisals Scale (SS-A)(84) | 23-item scale measures  perceived social support from family,  friends, and other members of the  immediate community. | Social support | 1 | Not reported | N/A |
| Social Support Index(85) | Scale includes completed measures of the amount  and quality of social support available to them from 20 different individuals (e.g., mother, father, coach, and best friend) and groups (e.g., their  teammates and clubs or religious groups to which they belonged). | Social support | 1 | Not reported | N/A |
| Sport Anxiety Scale (SAS)(86) | 21-item measure  of trait anxiety. It has separate subscales for somatic  anxiety, and for two varieties of cognitive anxiety: worry, and concentration  disruption. | Performance anxiety | 1 | Internal consistency reported in a cohort of professional ballet dancers.(86)  Not evaluated in musicians, vocalists or circus performers. | Internal consistency: Cronbach's α coefficients were 0.84 for somatic anxiety, 0.79 for Worry, and  0.76 for concentration disruption. |
| Sport Multidimensional Perfectionism Scale-2 (S-MPS-2)(87) | Six domains for evaluating the level of perfectionism regarding :personal standards ; concern  over mistakes;  doubts about action;  perceived parental pressure; perceived coach pressure; and  need for organisation | Perfectionism | 1 | Not reported | N/A |
| Stress and Coping Inventory (SCI)(88) | A self-report tool used to determine the current stress load and stress symptoms and to illustrate how to deal with stress using five coping strategies. It comprises 10 scales with 54 items. | Coping with stress | 1 | Not reported | N/A |
| State-Trait Anxiety Inventory (STAI-T)(89) | 20-item scale used to measure state (20 items) and trait (20 items) anxiety. | Anxiety | 4 | Not reported | N/A |
| Tampa Scale for Kinesiophobia-11 (TSK-11) (Spanish version)(90) | TSK-11 consists  of two subscales, one related to fear of activity and  the other related to fear of harm. The final score can range  between 11 and 44 points, with higher scores indicating  greater perceived kinesiophobia. | Fear of activity/harm | 1 | Not reported | N/A |
| Traumatic Events Questionnaire (TEQ)(91) | A self-report 11-item dichotomously scored instrument that assesses exposure to 9 different traumatic events | Legacy of trauma | 1 | Test-retest reliability reported in a cohort of undergraduate student dancers.(92)  Not evaluated in musicians/circus performers. | Test-retest of TEQ was found to be highly stable over this time span, with a correlation of .95 (p < .001) between the two TEQ scores. |
| University of North Texas Musician Health Survey(93) | A comprehensive self-report questionnaire designed to assess physical and mental health issues, performance-related injuries, and wellness behaviours among musicians. | Holistic wellness | 1 | Not reported | N/A |

**Supplementary Table 2: Instrument details**

**References**

1. Krug SE. The Adult Personality Inventory. J Couns Dev. 1991;69(3):266-71.<https://doi.org/10.1002/j.1556-6676.1991.tb01501.x>.

2. Felitti VJ, Anda RF, Nordenberg D, Williamson DF, Spitz AM, Edwards V, et al. Relationship of Childhood Abuse and Household Dysfunction to Many of the Leading Causes of Death in Adults: The Adverse Childhood Experiences (ACE) Study. A J Prevent Med. 1998;14(4):245-58.<https://doi.org/10.1016/S0749-3797(98)00017-8>.

3. Saunders JB, Aasland OG, Babor TF, De La Fuente JR, Grant M. Development of the Alcohol Use Disorders Identification Test (AUDIT): WHO Collaborative Project on Early Detection of Persons with Harmful Alcohol Consumption-II. Addiction. 1993;88(6):791-804.<https://doi.org/10.1111/j.1360-0443.1993.tb02093.x>.

4. Means-Christensen AJ, Sherbourne CD, Roy-Byrne PP, Craske MG, Stein MB. Using five questions to screen for five common mental disorders in primary care: diagnostic accuracy of the Anxiety and Depression Detector. Gen Hosp Psychiatry. 2006;28(2):108-18.<https://doi.org/10.1016/j.genhosppsych.2005.08.010>.

5. Smith RE, Schutz RW, Smoll FL, Ptacek JT. Development and Validation of a Multidimensional Measure of Sport-Specific Psychological Skills: The Athletic Coping Skills Inventory-28. J Sport Exerc Psychol. 1995;17(4):379-98.10.1123/jsep.17.4.379.

6. Raedeke TD, Smith AL. Development and Preliminary Validation of an Athlete Burnout Measure. J Sport Exerc Psychol. 2001;23(4):281-306.10.1123/jsep.23.4.281.

7. Casanova MP, Reeves AJ, Baker RT. Psychometric Properties of a Modified Athlete Burnout Questionnaire in the Collegiate Athletics Setting. J Sport Rehab. 2023;32(5):581-9.10.1123/jsr.2022-0217.

8. Beck AT, Epstein N, Brown G, Steer RA. An inventory for measuring clinical anxiety: Psychometric properties. J Consult Clin Psychol. 1988;56(6):893-7.10.1037/0022-006X.56.6.893.

9. Beck A, Steer R, Brown G. Beck depression inventory–II. Corporation P. San Antonio, Texas.1996.

10. Hyvönen K, Pylvänäinen P, Muotka J, Lappalainen R. The Effects of Dance Movement Therapy in the Treatment of Depression: A Multicenter, Randomized Controlled Trial in Finland. Front Psychol. 2020. <https://doi.org/10.3389/fpsyg.2020.01687>

11. Pallesen S, Bjorvatn B, Nordhus IH, Sivertsen B, Hjørnevik M, Morin CM. A New Scale for Measuring Insomnia: The Bergen Insomnia Scale. Percept Mot Skills. 2008;107(3):691-706.10.2466/pms.107.3.691-706.

12. Tylka TL, Wood-Barcalow NL. The Body Appreciation Scale-2: Item refinement and psychometric evaluation. Body Image. 2015;12:53-67.<https://doi.org/10.1016/j.bodyim.2014.09.006>.

13. Nicholas J, Dimmock J, Alderson J, Donnelly C, Jackson B. Exploring the psychological and physiological outcomes of recreational pole dancing: a feasibility study. Circus: Arts, Life, Sciences. 2024;2(2).<https://doi.org/10.3998/circus.2801>.

14. Carver CS. You want to measure coping but your protocol’ too long: Consider the brief cope. Int J Behav Med. 1997;4(1):92-100.10.1207/s15327558ijbm0401_6.

15. Barrell GM. Coping strategies used by ballet dancers : effects of individual differences in competitive trait anxiety: University of Southern Queensland, Australia  2001. Available at: https://eprints.qut.edu.au/69372/1/Masters_Thesis_-_Gene_M._Barrell.pdf

16. Sierra M, Berrios GE. The Cambridge Depersonalisation Scale: a new instrument for the measurement of depersonalisation. Psychiatry Res. 2000;93(2):153-64.<https://doi.org/10.1016/S0165-1781(00)00100-1>.

17. Flett GL, Hewitt PL, Besser A, Su C, Vaillancourt T, Boucher D, et al. The Child–Adolescent Perfectionism Scale: Development, Psychometric Properties, and Associations With Stress, Distress, and Psychiatric Symptoms. J Psychoeduc Assess. 2016;34(7):634-52.10.1177/0734282916651381.

18. Pentith R, Louise Moss S, Lamb K, Edwards C. Perfectionism among Young Female Competitive Irish Dancers: Prevalence and Relationship with Injury Responses. J Dance Med Sci. 2021;25(2):152-8.10.12678/1089-313X.061521k.

19. Cohen S, Kamarck T, Mermelstein R. A global measure of perceived stress. J Health Soc Behav. 1983:385-96

20. Kulshreshtha M, Saraswathy KN, Babu N, Chandel S. Self-reported perceived stress, depression, and generalized anxiety disorder among Kathak dancers and physically active non-dancers of North India. Front Psychol. 2023;14. https://doi.org/10.3389/fpsyg.2023.1114377

21. Martens R, Vealey RS, Burton D. Competitive anxiety in sport. Champaign, IL. Human Kinetics. 1990

22. Ioannou CI, KlÄMpfl MK, Lobinger BH, Raab M, AltenmÜLler E. Psychodiagnostics: Classification of the Yips Phenomenon based on Musician’s Dystonia. Med Sci Sports Exerc. 2018;50(11). https://doi.org/10.1249/mss.0000000000001696

23. Kristensen TS, Borritz M, Villadsen E, Christensen KB. The Copenhagen Burnout Inventory: A new tool for the assessment of burnout. Work Stress. 2005;19(3):192-207.10.1080/02678370500297720.

24. Endler N, Parker JDA. Coping inventory for stressful situations. Toronto, ON, Canada: Multi-Health Systems. 1990

25. Thomson P, Jaque SV. Posttraumatic Stress Disorder and Psychopathology in Dancers. Med Probl Perform Art. 2015;30(3):157-62.10.21091/mppa.2015.3030.

26. Rosenstiel AK, Keefe FJ. The use of coping strategies in chronic low back pain patients: Relationship to patient characteristics and current adjustment. Pain. 1983;17(1):33-44.<https://doi.org/10.1016/0304-3959(83)90125-2>.

27. Judge TA, Erez A, Bono JE, Thoresen CJ. The core self-evaluations scale: Development of a measure. Pers Psychol. 2003;56(2):303. https://doi.org/10.1111/j.1744-6570.2003.tb00152.x

28. Rip B, Fortin S, Vallerand RJ. The relationship between passion and injury in dance students. J Dance Med Sci. 2006;10(1-2):14-20. http://dx.doi.org/10.1177/1089313X06010001-205

29. Lovibond PF, Lovibond SH. The structure of negative emotional states: comparison of the Depression Anxiety Stress Scales (DASS) with the Beck Depression and Anxiety Inventories. Behav Res Ther. 1995;33(3):335-43.10.1016/0005-7967(94)00075-u.

30. Gratz KL, Roemer L. Difficulties in Emotion Regulation Scale. J Clin Psychol. 2004.  26(1):41-54. https://psycnet.apa.org/doi/10.1037/t01029-000.

31. Carlson EB, Putnam FW. The dissociative experiences scale (DES-II). Psychoanal Inq. 2000;20(2):361-6. https://psycnet.apa.org/doi/10.1037/t86316-000

32. Garner D M. Eating Disorder Inventory-2 ; Professional Manual. Odessa, FL, USA.Psychological Assessment Resources. 1991

33. Fairburn CG, Beglin SJ. Eating disorder examination questionnaire. Cognitive behavior therapy and eating disorders. New York,. Guilford Press. 2008.

34. Waddell G, Newton M, Henderson I, Somerville D, Main CJ. A Fear-Avoidance Beliefs Questionnaire (FABQ) and the role of fear-avoidance beliefs in chronic low back pain and disability. Pain. 1993;52(2). [10.1016/0304-3959(93)90127-B](https://doi.org/10.1016/0304-3959(93)90127-b).

35. Fonseca DM, Bonfate G, Valle AL, Freitas SFT. Diagnosis by anamnesis of craniomandibular dysfunction. Rev Gaucha Odontol. 1994;4(1):23-32

36. Zawadzki B, Strelau J. Structure of personality: Search for a general factor viewed from a temperament perspective. Pers Individ Differ. 2010;49(2):77-82.<https://doi.org/10.1016/j.paid.2010.03.025>.

37. Fahrenberg J, Hampel R, Selg, H. (2021). Freiburg Personality Inventory (FPI-R). Primary data from the 2018 norming sample. (Version 1.0.0). Trier: Research Data Center at ZPID. https://doi.org/10.5160/psychdata.fgjn18pr30

38. Frost RO, Marten P, Lahart C, Rosenblate R. The dimensions of perfectionism. Cogn Ther Res. 1990;14(5):449-68.10.1007/BF01172967. https://psycnet.apa.org/doi/10.1007/BF01172967

39. Kenny DT, Davis P, Oates J. Music performance anxiety and occupational stress amongst opera chorus artists and their relationship with state and trait anxiety and perfectionism. J Anxiety Disord. 2004;18(6):757-77.<https://doi.org/10.1016/j.janxdis.2003.09.004>.

40. Goldberg DP, Hillier VF. A scaled version of the General Health Questionnaire. Psychol Med. 1979;9(1):139-45.10.1017/S0033291700021644.

41. Schwarzer R, Jerusalem M. Generalized self-efficacy scale. J Weinman, S Wright, & M Johnston, Measures in health psychology: A user’s portfolio Causal and control beliefs. 1995; 35(37):82-003. https://psycnet.apa.org/doi/10.1037/t00393-000

42. Spitzer RL, Kroenke K, Williams JBW, Löwe B. A Brief Measure for Assessing Generalized Anxiety Disorder: The GAD-7. Arch Intern Med. 2006;166(10):1092-7.10.1001/archinte.166.10.1092.

43. Derogatis LR, Lipman RS, Rickels K, Uhlenhuth EH, Covi L. The Hopkins Symptom Checklist (HSCL): A self-report symptom inventory. Behav Sci. 1974;19(1):1-15.<https://doi.org/10.1002/bs.3830190102>.

44. Vaag J, Bjørngaard JH, Bjerkeset O. Symptoms of anxiety and depression among Norwegian musicians compared to the general workforce. Psychol Music. 2015;44(2):234-48.10.1177/0305735614564910.

45. Zigmond AS, Snaith RP. The Hospital Anxiety and Depression Scale. Acta Psychiatr Scand. 1983;67(6):361-70.<https://doi.org/10.1111/j.1600-0447.1983.tb09716.x>.

46. Musgrave G, Gross SA, Carney D. Determinants of Anxiety, Depression and Subjective Wellbeing Among Musicians in Denmark: Findings From the ‘When Music Speaks’ Project. Scand J Psychol. 2025;n/a(n/a).<https://doi.org/10.1111/sjop.13095>.

47. Parker DF, DeCotiis TA. Organizational determinants of job stress. Organ Behav Hum Perform. 1983;32(2):160-77.<https://doi.org/10.1016/0030-5073(83)90145-9>.

48. Karasek Jr RA. Job demands, job decision latitude, and mental strain: Implications for job redesign. Adm Sci Q. 1979:285-308. DOI: 10.2307/2392498

49. Kenny DT. The Kenny music performance anxiety inventory (K-MPAI): Scale construction, cross-cultural validation, theoretical underpinnings, and diagnostic and therapeutic utility. Front Psychol. 2023;14. https://doi.org/10.3389/fpsyg.2023.1143359

50. Kessler RC, Barker PR, Colpe LJ, Epstein JF, Gfroerer JC, Hiripi E. Kessler psychological distress scale (K10). Boston, MA: Harvard Medical School. 1996

51. Sarason IG, Johnson JH, Siegel JM. Assessing the impact of life changes: Development of the Life Experiences Survey. J Consult Clin Psychol. 1978;46(5):932-46.10.1037/0022-006X.46.5.932.

52. Veit CT, Ware JE. The structure of psychological distress and well-being in general populations. J Consult Clin Psychol. 1983;51(5):730-42.10.1037/0022-006X.51.5.730.

53. Zábó V, Oláh A, Vargha A. A new complex mental health test in a positive psychological framework. Front Psychol. 2022;13.10.3389/fpsyg.2022.775622.

54. Weiss DJ, Dawis RV, England GW, Lofquist LH. Minnesota satisfaction questionnaire--short form. Educ Psychol Meas. 1977. https://psycnet.apa.org/doi/10.1037/t08880-000

55. Multiple Sclerosis Council for Clinical Practice Guidelines. Fatigue and multiple sclerosis: evidence-based management strategies for fatigue in multiple sclerosis: clinical practice guidelines. Washington DC. America PVo. Paralyzed Veterans of America; 1998.

56. Berque P, Gray H, McFadyen A. Development and psychometric evaluation of the Musculoskeletal Pain Intensity and Interference Questionnaire for professional orchestra Musicians. Man Ther. 2014;19(6):575-88.<https://doi.org/10.1016/j.math.2014.05.015>.

57. Conley KM, Bolin DJ, Carek PJ, Konin JG, Neal TL, Violette D. National Athletic Trainers' Association Position Statement: Preparticipation Physical Examinations and Disqualifying Conditions. J Athl Train. 2014;49(1):102-20.10.4085/1062-6050-48.6.05.

58. Hurrell Jr JJ, Worthington KA, Driscoll RJ. Job stress, gender, and workplace violence: analysis of assault experiences of state employees. Violence on the job: Identifying risks and developing solutions. Washington, DC, US: American Psychological Association; 1996. p. 163-70.

59. McCrae RR. The Five-Factor Model and Its Assessment in Clinical Settings. J Pers Assess. 1991;57(3):399-414.10.1207/s15327752jpa5703_2.

60. Osipow SH, Spokane AR. A manual for measures of occupational stress, strain, and coping:(Form E-2). Odessa Florida, United States. Marathon Consulting and Press; 1983.

61. Linton SJ, Boersma K. Early Identification of Patients at Risk of Developing a Persistent Back Problem: The Predictive Validity of The Örebro Musculoskeletal Pain Questionnaire. Clin J Pain. 2003;19(2) 80-86.

62. McCracken LM, Dhingra L. A Short Version of the Pain Anxiety Symptoms Scale (PASS-20): Preliminary Development and Validity. Pain Res Manag. 2002;7(1):517163.<https://doi.org/10.1155/2002/517163>.

63. Sullivan MJL, Bishop SR, Pivik J. The Pain Catastrophizing Scale: Development and validation. Psychol Assess. 1995;7(4):524-32.10.1037/1040-3590.7.4.524.

64. Vallerand RJ, Blanchard C, Mageau GA, Koestner R, Ratelle C, Léonard M, et al. Les passions de l'âme: On obsessive and harmonious passion. J Pers Soc Psychol. 2003;85(4):756-67.10.1037/0022-3514.85.4.756.

65. Spitzer RL, Kroenke K, Williams JBW, and the Patient Health Questionnaire Primary Care Study G. Validation and Utility of a Self-report Version of PRIME-MDThe PHQ Primary Care Study. JAMA. 1999;282(18):1737-44.10.1001/jama.282.18.1737.

66. Compas BE, Davis GE, Forsythe CJ, Wagner BM. Assessment of major and daily stressful events during adolescence: the Adolescent Perceived Events Scale. J Consult Clin Psychol. 1987;55(4):534. https://psycnet.apa.org/doi/10.1037/0022-006X.55.4.534

67. Nordin-Bates SM, Cumming J, Aways D, Sharp L. Imagining Yourself Dancing to Perfection? Correlates of Perfectionism Among Ballet and Contemporary Dancers. J Clin Sport Psychol. 2011;5(1):58-76.10.1123/jcsp.5.1.58.

68. Cox WJ, Kenardy J. Performance anxiety, social phobia, and setting effects in instrumental music students. J Anxiety Disord. 1993;7(1):49-60.<https://doi.org/10.1016/0887-6185(93)90020-L>.

69. Lamontagne V, Bélanger C. Pain-related and performance anxiety and their contribution to pain in music students: a pilot study. Health Psycho Rep. 2015;3(1):59-68.10.5114/hpr.2015.47088.

70. Brandt PA, Weinert C. The PRQ--a social support measure. Nurs Res. 1981;30(5):277-80

71. Buysse DJ, Reynolds CF, Monk TH, Berman SR, Kupfer DJ. The Pittsburgh sleep quality index: A new instrument for psychiatric practice and research. Psychiatry Res. 1989;28(2):193-213.<https://doi.org/10.1016/0165-1781(89)90047-4>.

72. Watson D, Clark LA, Tellegen A. Development and validation of brief measures of positive and negative affect: the PANAS scales. J Pers Soc Psychol. 1988;54(6):1063-70.10.1037//0022-3514.54.6.1063.

73. McNair DM, Lorr M, Droppleman LF. Manual profile of mood states. San Diego, United States. Educational & Industrial Testing Service.1971

74. Krampen G. Fragebogen zu Kompetenz-und Kontrollüberzeugungen:(FKK): Hogrefe, Göttingen. Verlag für Psychologie; 1991.

75. Kellmann M, Kallus KW. Recovery-stress questionnaire for athletes: User manual. Champaign, IL, US: Human Kinetics; 2001. xi, 73-xi, p.

76. Silva LO, Mendes LMR, Lima POD, Almeida GPL. Translation, cross-adaptation and measurement properties of the Brazilian version of the ACL-RSI Scale and ACL-QoL Questionnaire in patients with anterior cruciate ligament reconstruction. Braz J Phys Ther. 2018;22(2):127-34.10.1016/j.bjpt.2017.09.006.

77. Wagnild G M, Young H M. Development and psychometric evaluation of the Resilience Scale. J Nurs Meas. 1993. 1(2), 165–178.

78. Rosenberg M. Society and the Adolescent Self-Image: Princeton, New Jersey, United States. Princeton University Press; 1965.

79. Carey KB, Neal DJ, Collins SE. A psychometric analysis of the self-regulation questionnaire. Addict Behav. 2004;29(2):253-60.10.1016/j.addbeh.2003.08.001.

80. Strand BH, Dalgard OS, Tambs K, Rognerud M. Measuring the mental health status of the Norwegian population: A comparison of the instruments SCL-25, SCL-10, SCL-5 and MHI-5 (SF-36). Nord J Psychiatry. 2003;57(2):113-8.10.1080/08039480310000932.

81. Ware JE, Jr., Sherbourne CD. The MOS 36-ltem Short-Form Health Survey (SF-36): I. Conceptual Framework and Item Selection. Med Care. 1992;30(6). 473-83.

82. Morgan JF, Reid F, Lacey JH. The SCOFF questionnaire: assessment of a new screening tool for eating disorders. BMJ. 1999;319(7223):1467.10.1136/bmj.319.7223.1467.

83. Connor KM, Davidson JRT, Churchill LE, Sherwood A, Weisler RH, Foa EB. Psychometric properties of the Social Phobia Inventory (SPIN). Brit J Psychiatry. 2000;176:379 - 86.10.1192/bjp.176.4.379.

84. Vaux A, Phillips J, Holly L, Thomson B, Williams D, Stewart D. The social support appraisals (SS-A) scale: Studies of reliability and validity. Am J Community Psychol. 1986;14(2):195-219.10.1007/BF00911821.

85. Smith RE, Smoll FL, Ptacek JT. Conjunctive moderator variables in vulnerability and resiliency research: Life stress, social support and coping skills, and adolescent sport injuries. J Pers Soc Psychol. 1990;58(2):360-70.10.1037/0022-3514.58.2.360.

86. Smith RE, Smoll FL, Schutz RW. Measurement and correlates of sport-specific cognitive and somatic trait anxiety: The Sport Anxiety Scale. Anxiety Res. 1990;2(4):263-80.10.1080/08917779008248733.

87. Skwiot M, Śliwiński Z, Śliwiński GE. Perfectionism and Burnout in Sport and Dance. Phys Med Rehabil Kurortmed. 2020;30(03):135-40.10.1055/a-1089-8125.

88. Satow, L. SCI – Stress- und Coping-Inventar. Stress and coping inventory [questionnaire with examples and scale documentation]. In Leibniz Center for Psychological Information and Documentation (ZPID). (ed.), Electronic Test Archive (PSYNDEX Test No. 9006508). https://doi.org/10.23668/PSYCHARCHIVES.424. 2012.

89. Spielberger CD, Gonzalez-Reigosa F, Martinez-Urrutia A, Natalicio LFS, Natalicio DS. The State-Trait Anxiety Inventory. Interam J Psychol. 2017;5(3 & 4).10.30849/rip/ijp.v5i3 & 4.620.

90. Gómez-Pérez L, López-Martínez AE, Ruiz-Párraga GT. Psychometric Properties of the Spanish Version of the Tampa Scale for Kinesiophobia (TSK). J Pain. 2011;12(4):425-35.10.1016/j.jpain.2010.08.004.

91. Lauterbach D, Vrana S. The Relationship Among Personality Variables, Exposure to Traumatic Events, and Severity of Posttraumatic Stress Symptoms. J Trauma Stress. 2001;14(1):29-45.10.1023/A:1007831430706.

92. Thomson P, Jaque SV. Anxiety and the Influences of Flow, Trauma, and Fantasy Experiences on Dancers. Imagin Cogn Pers. 2012;32(2):165-78.10.2190/IC.32.2.e.

93. Pak CH, Chesky K. Prevalence of Hand, Finger, and Wrist Musculoskeletal Problems in Keyboard Instrumentalists: The University of North Texas Musician Health Survey. Med Probl Perform Arts. 2001;16(1):17-23.10.21091/mppa.2001.1004.
